# Tendon extracellular-matrix-derived tissue engineering micro-tissue for Achilles tendon injury regeneration in rats

**DOI:** 10.1186/s13018-024-04863-0

**Published:** 2024-06-27

**Authors:** Kaihong Zhang, Peng Zhang, Ge Shi, Lei Wang, Chen Sun, Wei Xiang

**Affiliations:** 1 Department of Orthopedics, Wuhan Jinyintan Hospital, Tongji Medical College of Huazhong University of Science and Technology, Wuhan, China; 2 Department of Orthopedics, Renmin Hospital of Wuhan University, Wuhan University, Wuhan, China

**Keywords:** Rat-tail tendon, Extracellular matrix, Tendon repair, Achilles tendon, Tissue engineering

## Abstract

**Background:**

Achilles tendon is vital in maintaining the stability and function of ankle joint. It is quite difficult to achieve the structural and functional repair of Achilles tendon in tissue engineering.

**Methods:**

A tissue-engineered tendon micro-tissue was prepared using rat tail tendon extracellular matrix (TECM) combined with rat adipose stem cells (ADSCs) to repair Achilles tendon injuries. The TECM was prepared by repeated freezing and thawing. The in vitro characteristics of TECM and its effect on ADSCs proliferation were detected. This tissue-engineered tendon micro-tissue for Achilles tendon repair in vivo was evaluated based on general characteristics, gait analysis, ultrasound findings, histological analysis, and biomechanical testing.

**Results:**

The results showed that the TECM scaffold had good biocompatibility for ADSCs. At 2 weeks post-surgery, collagen types I and III and tenomodulin expression were higher, and vascular endothelial growth factor expression was lower in the micro-tissue group than other groups. At 4 and 8 weeks post-surgery, the results of histological analysis and ultrasound findings showed that the repaired tendon tissue was smooth and lustrous, and was arranged regularly and evenly in the micro-tissue group. Gait analysis confirmed that better motor function recovery was noted in micro-tissue group than other groups. In addition, the mechanical properties of the repaired tendon tissue in micro-tissue group were better than other groups.

**Conclusion:**

Tissue-engineered tendon micro-tissue fabricated by TECM and ADSCs has good biocompatibility and can promote structural and functional repair of tendon in vivo. This composite biomaterial has broad application prospects in tissue engineering.

## Background

The Achilles tendon is an important connective tissue that maintains joint stability and normal movement. Due to the lack of vascularization and therefore the lack of nutrients, the ability of Achilles tendon to repair itself is poor [1]. In addition, an Achilles tendon injury can lead to complications related to motor disorders such as pain, joint instability, cartilage damage, and ultimately osteoarthritis. These complications can seriously affect the normal function of limbs and joints. Previous studies reported that more than 30 million cases of tendon and ligament damage occur worldwide every year [2], and the incidence of Achilles tendon rupture was in the range of (11–37)/10^5^ [3].

Traditional treatments for Achilles tendon injuries include surgical, non-surgical and rehabilitation approaches. The choice of treatment depends on the severity of the Achilles tendon injury. For example, complete or open Achilles tendon ruptures often require surgical treatment. Surgical interventions include direct suture, xenograft or allograft. Conservative treatment can be chosen for Achilles tendon injuries with partial rupture and no functional impairment [4, 5]. Due to poor vascularization, limited cellularity, and strong fibrous adhesion during the healing process, it is difficult to achieve fully functional repair of the tendon with current treatments. In addition, fibrotic scar formation is the most common form of Achilles tendon healing. The formation of scar tissue leads to a reduction in the biomechanical properties of the Achilles tendon, such as mechanical strength and elasticity, leading to an increased risk of re-rupture [5–7]. A study reported that the re-rupture rate of the Achilles tendon was 1.7–10% higher after conservative treatment than after surgical treatment, but there was no risk of incision infection or nerve injury with conservative treatment [8]. In addition, surgery can also lead to joint stiffness and poor healing effects. Subsequent re-rupture is not uncommon because it is very difficult to completely restore a damaged tendon to its pre-injury condition [9]. Therefore, improving tendon healing and restoring its normal function remain the major challenges.

In tendon tissue engineering, the creation of a suitable scaffold is a crucial aspect. The ideal scaffold should have good biocompatibility with the host, provide a suitable environment for seed cells growth and differentiation, and have the required biomechanical properties [10]. Animal experiments and clinical studies have shown that the extracellular matrix (ECM) has reduced tissue immunity and good biocompatibility with host and seed cells and restores the function of damaged tissue [11]. Furthermore, ECM provided seed cells with a microenvironment that simulated the microstructure of tissue in situ and played an important role in maintaining stem cell properties [12]. In this study, we constructed a kind of tissue-engineered tendon micro-tissue by combining rat tail tendon extracellular matrix (TECM) with rat adipose stem cells (ADSCs). We then investigated the application of this composite scaffold in the repair of Achilles tendon injuries in rats.

## Methods

### Preparation and characterization of TECM

#### Preparation of TECM

20 Sprague–Dawley (SD) rats were first anesthetized and then sacrificed to obtain the tails. The tails of the rats were separated at the articular segment. The tail tendon was completely extracted, washed thoroughly with phosphate-buffered saline (PBS), and soaked in hydrogen peroxide overnight. The tissue was then subjected to five freeze–thaw cycles consisting of 10 min at − 80 °C in a freezer and 10 min at 37 °C in a thermostatically controlled water bath. After thoroughly rinsing with sterile PBS, the tendon was decellularized by sterilely incubating the tissue in 1% SDS at 37 °C on a shaker for 24 h. After several 3 h washes in PBS for a week, TECM was obtained [13, 14].

#### General and histological observations of TECM

The morphology of TECM was observed by camera. The hematoxylin–eosin (HE) and immunofluorescence staining assays were used to evaluate the decellularization of TECM. The detailed procedures were referred to previous study.

#### Determination of residual α-gal content in TECM

Detection of α-gal content was used to evaluate the immunogenicity of TECM after decellularization. The antibody of α-gal was added to the appropriate wells according to the kit manufacturer’s instructions. The wells were sealed with a sealing membrane and placed in an incubator at 37 °C for 60 min. The sealing membrane was then carefully removed and the wells were washed with buffer. Enzyme labeling reagent (100 µl) was then added to each well for 30 min. After the wells were washed, equal volumes of solutions A and B were added, followed by 100 µl of dye. The plate was incubated in the dark for 30 min, after which 50 µl of termination solution was quickly added to each well with gentle oscillation. The optical density of 100 µl of the sample or standard was then measured at an absorbance of 450 nm [15].

#### Detection of biocompatibility with ADSCs

100 µl cell suspension was incubated in a 96-well plate for 24 h (37 °C, 5% CO_2_). The leaching solution was prepared according to the instructions of the CCK-8 reagent kit [16], and 10 µl was added to each well. The plate was then incubated for 1–6 days, after which 10 µl of CCK-8 solution was added to each well. After 2 h of incubation, the absorbance was determined at 450 nm.

### Isolation, culture, and identification of ADSCs and the construction of tissue-engineered tendon micro-tissue

#### Isolation and culture of ADSCs

The fat in the inguinal area was harvested under aseptic conditions from two 4-week-old green fluorescent protein (GFP) SD rats. The cell suspension was prepared as previously described [17]. The isolated GFP-ADSCs were cultured in growth medium (DMEM-low glucose, 10% fetal bovine serum (FBS), 100 U penicillin/ml and 100 mg streptomycin/ml, Corning) and subcultured when they reached 80–90% confluence. The medium was changed every 3 days.

#### Identification of ADSCs

A suspension of passage 2 (P2) cells at a concentration of 1 × 10^6^/ml was incubated with CD29, CD34, CD45, and CD71 monoclonal antibodies for 30 min at room temperature, after which antigen expression was detected by flow cytometry.

#### Adipogenic, osteogenic, and cartilage differentiation of ADSCs

P2-ADSCs were inoculated into six-well plates containing conditional medium to induce adipogenic, osteogenic and cartilage differentiation. The cells were then stained with oil red O, alizarin red, and alcian blue to confirm their ability to multi-directionally differentiate.

#### Co-culture and observation of ADSCs on TECM

An appropriate amount of sterile TECM was placed in the wells of a six-well plate, and 3 × 10^6^ P2 ADSCs were added. Cells were cultured in growth medium (DMEM-low glucose, 10% FBS, 100 U penicillin/ml and 100 mg streptomycin/ml, Corning) at 37 °C and 5% CO_2_. After 1 and 7 days, the cell-TECM complex was removed for Live-dead staining and observation with a laser confocal microscope.

### Tissue-engineered tendon micro-tissue implantation within a rat Achilles tendon repair model

#### Animal models and experimental groups

Forty-eight healthy male and female SD rats weighing 200–220 g were randomly divided into four groups of twelve individuals according to the design principles of randomized controlled trials. The rats were shaved, disinfected after anesthesia, and placed prone on the operating table. A 1 cm skin incision was made on the right hind leg of the Achilles tendon, carefully separating the fascia to open the tendon sheath. The transverse section of the Achilles tendon, approximately 5 mm above the root bone, was exposed. The blank group underwent surgery without treatment. The cell group was injected with ADSCs. The micro-carrier group was treated with TECM. The micro-tissue group with tissue-engineered tendon micro-tissue. GFP expression in the defect area was observed 2 weeks after surgery, and tissues from three rats per group were used for PCR. Ultrasound and gait analysis were performed at 4 and 8 weeks. Biomechanical tests and HE and Masson trichrome staining were performed on three rats in each group.

#### RT-PCR

Two weeks after surgery, the new tissue was removed under aseptic conditions and the expression of collagens type I (Col I), type III (Col III), fibrin (FBN), VEGF, and tenomodulin (TNMD) was analyzed as follows [18]: 1 ml of Trizol reagent (Invitrogen, USA) was added to a homogenate tube and cooled on ice, after which 100 mg of tissue was added. The mixture was completely homogenized and then total RNA was extracted according to the kit manufacturer’s instructions [19]. After reverse transcription, PCR was performed using the primer sequences listed in Table 1. The relative expression level of each target gene was calculated using the 2^−△△Ct^ method. The data was then statistically analyzed.

#### Gait analysis

The catwalk gait analysis method was used to evaluate the rat’s gait parameters along with the recovery of limb activity [20]. At 2, 4 and 8 weeks post-surgery, five rats were randomly selected from each group and their gait was analyzed for mean intensity and footprint area as indicators of recovery of the affected limbs.

#### Ultrasound

At 4 and 8 weeks post-surgery, three rats in each group were anesthetized. The hair on the posterior skin of the Achilles tendon was shaved and the healing of the tendon was assessed using ultrasound. A longitudinal examination of the Achilles tendon was performed and the rats’ ankle were subjected to passive flexion. The movement of the injured Achilles tendon was examined dynamically and the results were compared with those of the healthy side [21].

#### Histological analysis

At 4 and 8 weeks post-surgery, the Achilles tendon tissue was fixed in 4% paraformaldehyde for 48 h and then embedded in paraffin after dehydration. Sections of 7 μm were prepared for HE and Masson trichrome staining. Achilles tendon repair was assessed using histological staining results and scored using the Bonar scale, with a lower score indicating greater similarity to normal tissue [22]. Pathological images (including multiple areas of the repair edge to the center) were evaluated by three observers.

#### Biomechanical testing

At 8 weeks post-surgery, three specimens from each group were fixed at both ends of a mechanical loading device (Exlar-GSX, Germany). Before loading, ten preloads of 0–5 N were performed to eliminate the viscoelasticity of the Achilles tendon. The specimen was longitudinal loaded at 1 mm/min, and the force–displacement curve was recorded. The maximum load and tensile strength were also determined. The ultimate load (N) was defined as the maximum load before Achilles tendon rupture. Tensile strength (MPa) was defined as the maximum load divided by the cross-sectional area as previously described [17, 23–25].

#### Statistical analysis

These data were described as means ± standard deviations and analyzed by one-way ANOVA and post-hoc Tukey tests for multiple comparisons, and by two-tailed unpaired Student’s t-tests for pair-wise comparisons. Statistical analyzes were performed using SPSS Statistics 17.0 statistical software package. A *p* value < 0.05 was considered to indicate statistical significance. The significance level is presented as *p < 0.05 or **p < 0.01.

## Results

### General characteristics and histological observation of TECM

**Fig. 1:**
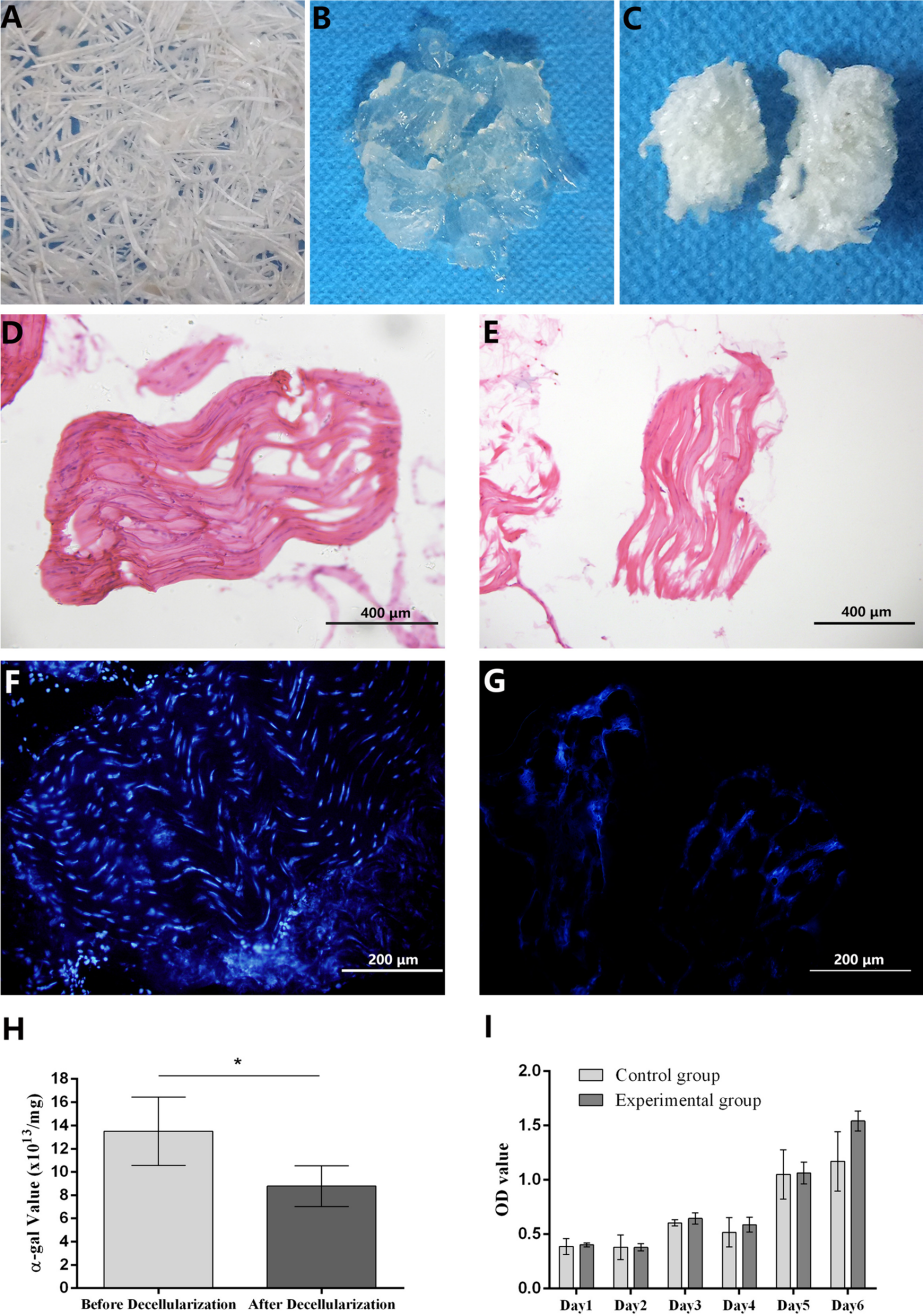
General characteristics of TECM. **A** Tendon before decellularization. **B** Tendon after decellularization. **C** TECM after drying and freezing. **D** HE staining before decellularization (magnification × 100, scale bar = 400 μm). **E** HE staining after decellularization (magnification × 100, scale bar = 400 μm). **F** DAPI staining before decellularization (magnification × 200, scale bar = 200 μm). **G** DAPI staining after decellularization (magnification × 200, scale bar = 200 μm). **H** Result of TECM residues α-gal content detection (*p < 0.05). **I** Result of biocompatibility detection with ADSCs

As shown in Fig. 1, the general characteristics of the tendon fiber before decellularization were a white stripe with complete structure and smooth surface (Fig. 1A). After decellularization, the material became more transparent, increased in volume, and had a gelatinous rather than bundle-like shape (Fig. 1B). The TECM scaffold was prepared after drying and freezing and had a white fluffy structure (Fig. 1C). To compare the microstructural changes during preparation, the histological HE staining test was used. The results of HE staining showed the tight arrangement of the tendon with a complete structure and cells arranged in a beaded pattern before decellularization (Fig. 1D). After decellularization, the structure of the tendon was loose, the volume had increased, and almost no tenocytes remained in this material (Fig. 1E). Furthermore, the result of DAPI staining showed a large number of blue-stained, closely aligned nuclei in the tendon tissue before decellularization (Fig. 1F), while only a small amount of nuclear residue remained in the tissue after decellularization (Fig. 1G).

To investigate the immunogenicity and biocompatibility of this material after decellularization, the α-gal content and cell viability of TECM residues were determined. The result of α-gal content detection showed that TECM samples after decellularization have significantly lower α-gal content in the caudal tendon than before decellularization (Fig. 1H, *p* < 0.05). Furthermore, ADSCs were seeded into materials to evaluate their cytotoxicity and biocompatibility. During the incubation period, the OD value showed an increasing trend, but the difference between control group and TECM group was not statistically significant (*p* > 0.05). The result showed that the TECM scaffold had promoting effects on ADSCs proliferation. This material has no obvious cytotoxicity and good biocompatibility for cell growth (Fig. 1I).

### The morphology and multi-differentiation potential of ADSCs

**Fig. 2:**
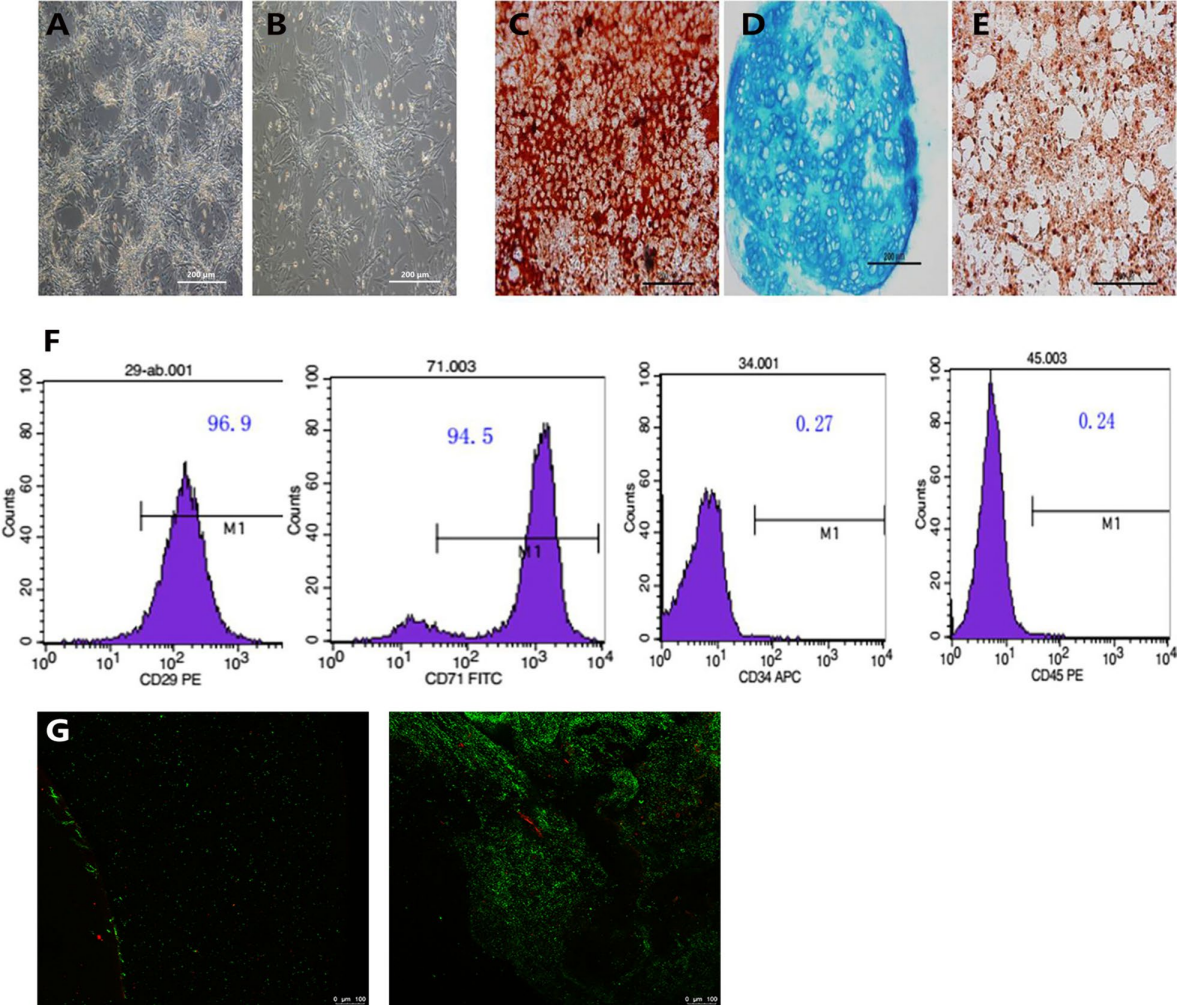
The morphology and multi-differentiation potential of ADSCs. **A** The morphology of P1 ADSCs (magnification × 100, scale bar = 200 μm). **B** The morphology of P2 ADSCs (magnification × 100, scale bar = 200 μm). **C**–**E** Multi-differentiation potential of ADSCs (magnification × 100, scale bar = 200 μm). **F** The results of flow cytometry **G** TECM co-cultured with ADSCs for 1 day. **H** TECM co-cultured with ADSCs for 7 days (magnification × 100, scale bar = 100 μm)

The morphology of ADSCs was observed under the microscope. During 24 h of cultivation, adipose tissue cells gradually adhered to the well and acquired a variety of forms. After 48 h, the ADSCs were passaged. P1 ADSCs were polygonal or long spindle-shaped cells with clear outlines and whirlpool-like growth (Fig. 2A). At 80–90% confluence, the cells were passaged again. P2 cells were uniform, long, spindle-shaped, and densely arranged (Fig. 2B). Flow cytometry was then used to detect stem cells biomarkers. The result of flow cytometry showed that more than 96.9% and more than 94.5% of ADSCs were positive for the stem cell markers CD29 and CD71, respectively, whereas less than 1% of the cells expressed the hematopoietic stem cell markers CD34 and CD45 (Fig. 2F). These results confirmed the potential of the cultured cells to follow multiple differentiation lines. We further validated the multidirectional differentiation ability of ADSCs. The results showed that some calcium nodules were observed in the cells after 10 days of osteogenic induction. Three weeks later, the nodules had enlarged and the cells had formed clumps that stained positive with alizarin red (Fig. 2C). Three days after the induction of chondrogenesis, the cells gradually aggregated into spheres. After 4 weeks, the spheres had become larger. Alcian blue staining revealed the presence of mucopolysaccharides in the cartilage tissue (Fig. 2D). After 3 days of culture in fat-inducing medium, the ADSCs gradually became long and spindle-shaped, with some lipid droplets appearing in the cells. After 8 days of induction, lipid droplets that stained positively with oil red O appeared in a large number of cells (Fig. 2E). Furthermore, ADSCs were co-cultured with TECM. A large number of live, highly active cells were attached on day 1, and only a few dead cells were seen (Fig. 2G). With increasing culture time, ADSCs continued to expand, with cell activity increasing significantly after 7 days, but the number of dead cells did not change significantly (Fig. 2H).

### Analysis of phenotypic genes expression of tissue-engineered tendon

**Fig. 3:**
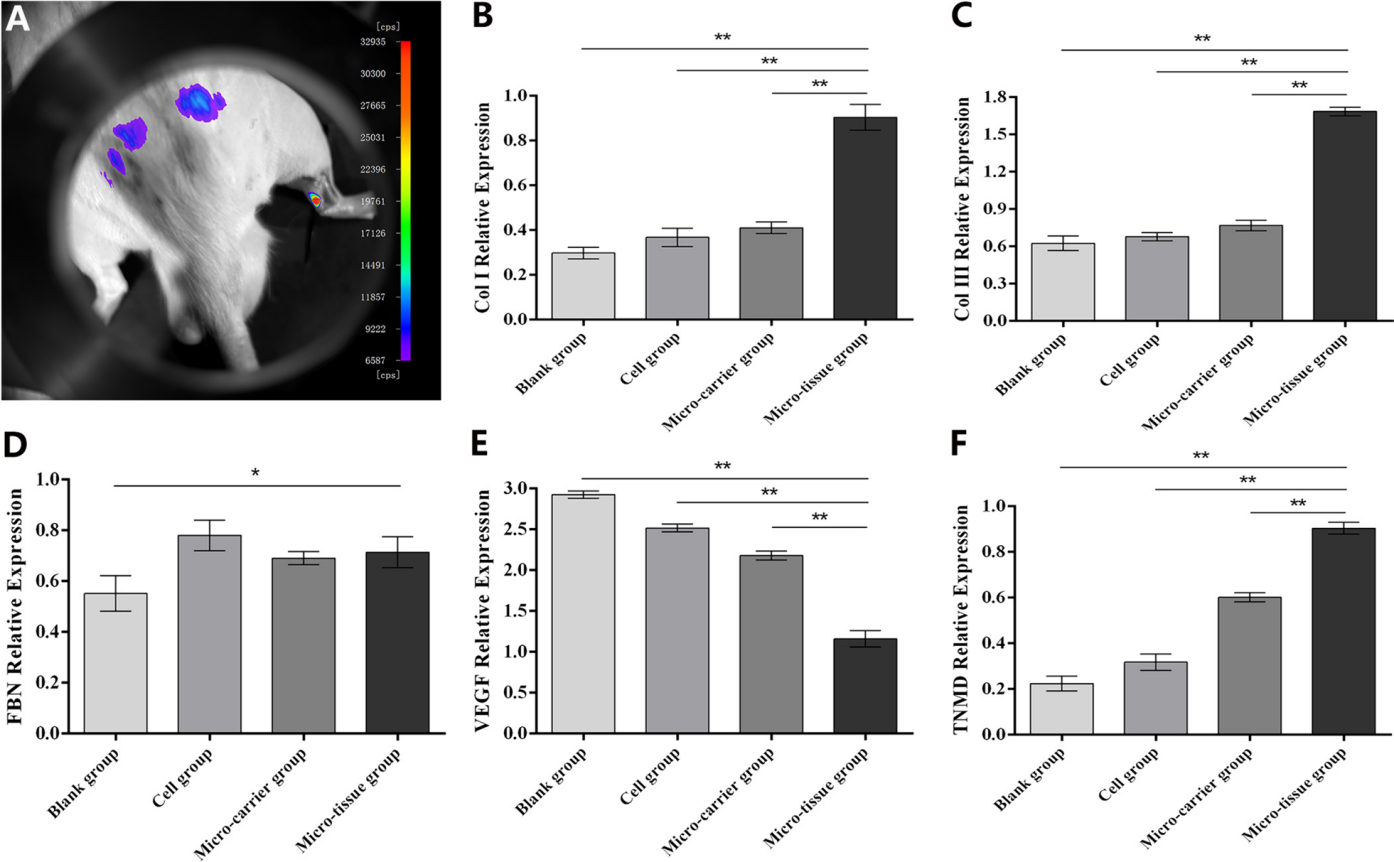
Observation of GFP fluorescence and analysis of phenotypic genes expression of tissue-engineered tendon. **A** Observation of GFP fluorescent signal in defect area at 2 weeks. **B**–**F** The expression of tendon-associated factors at 2 weeks. (*p < 0.05 and **p < 0.01)

To determine the survival of ADSCs in the defect area, the transplanted ADSCs were labeled with a GFP fluorescent signal. 2 weeks after surgery, GFP-labeled ADSCs still showed high signal expression in the defect area (Fig. 3A). To investigate the differentiation ability of tissue-engineered tendons in vivo, phenotypic genes such as Col I, Col III, FBN, VEGF and TNMD were analyzed by RT-PCR. The results showed that 2 weeks after surgery, Col I expression in the micro-tissue group was higher than in the other groups (*p* < 0.01) (Fig. 3B). Col III expression was significantly higher in the micro-tissue group than in the other groups (*p* < 0.01) (Fig. 3C). FBN expression in the micro-tissue, cell, and micro-carrier groups was not significantly different (*p* > 0.05). However, FBN expression in the micro-tissue group was significantly higher than that in the blank group (*p* < 0.05) (Fig. 3D). Two weeks after surgery, VEGF expression in the micro-tissue group was lower than that in the other groups (*p* < 0.01) (Fig. 3E). In the micro-tissue group, TNMD expression was significantly higher than in the other groups (*p* < 0.01) (Fig. 3F).

### Result of gait analysis, mean intensity, and footprint area

**Fig. 4:**
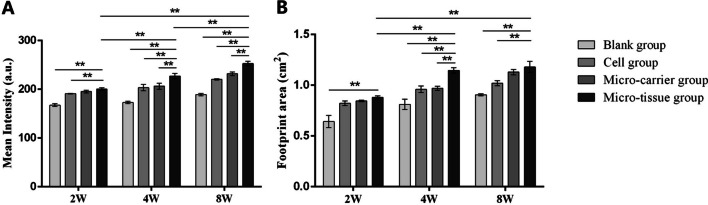
Result of gait analysis. **A** Mean intensity of affected limbs at 2, 4 and 8 weeks. **B** Footprint area of affected limbs at 2, 4 and 8 weeks (**p < 0.01)

At 2 weeks post-surgery, rats in each group had different degrees of claudication, and even in the blank group, rats were unable to extend their limbs to the ground. At this time, the mean intensity (Fig. 4A) of the affected limb was similar in all groups, but the mean intensity of the micro-tissue group was greater than that of the other groups. In particular, the micro-tissue group differed significantly from the blank and cell groups (*p* < 0.05) but not from the micro-carrier group (*p* > 0.05). Overall, the difference between the micro-tissue group and the other groups at 4 and 8 weeks was statistically significant (*p* < 0.05). In the micro-tissue group, the mean intensity of the affected limbs gradually increased (*p* < 0.05) at 2, 4 and 8 weeks. Even 2 weeks after surgery, the footprint area (Fig. 4B) of the micro-tissue group was larger than that of the blank group (*p* < 0.05) but not significantly different from that of the micro-carrier and cell groups (*p* > 0.05). At 4 and 8 weeks, the footprint area of the micro-tissue group was also significantly larger than that of other groups (*p* < 0.05). Over time, the footprint area of the micro-tissue group increased significantly. Taken together, these results showed that the micro-tissue group had the best recovery of motor function.

### Ultrasound findings

**Fig. 5:**
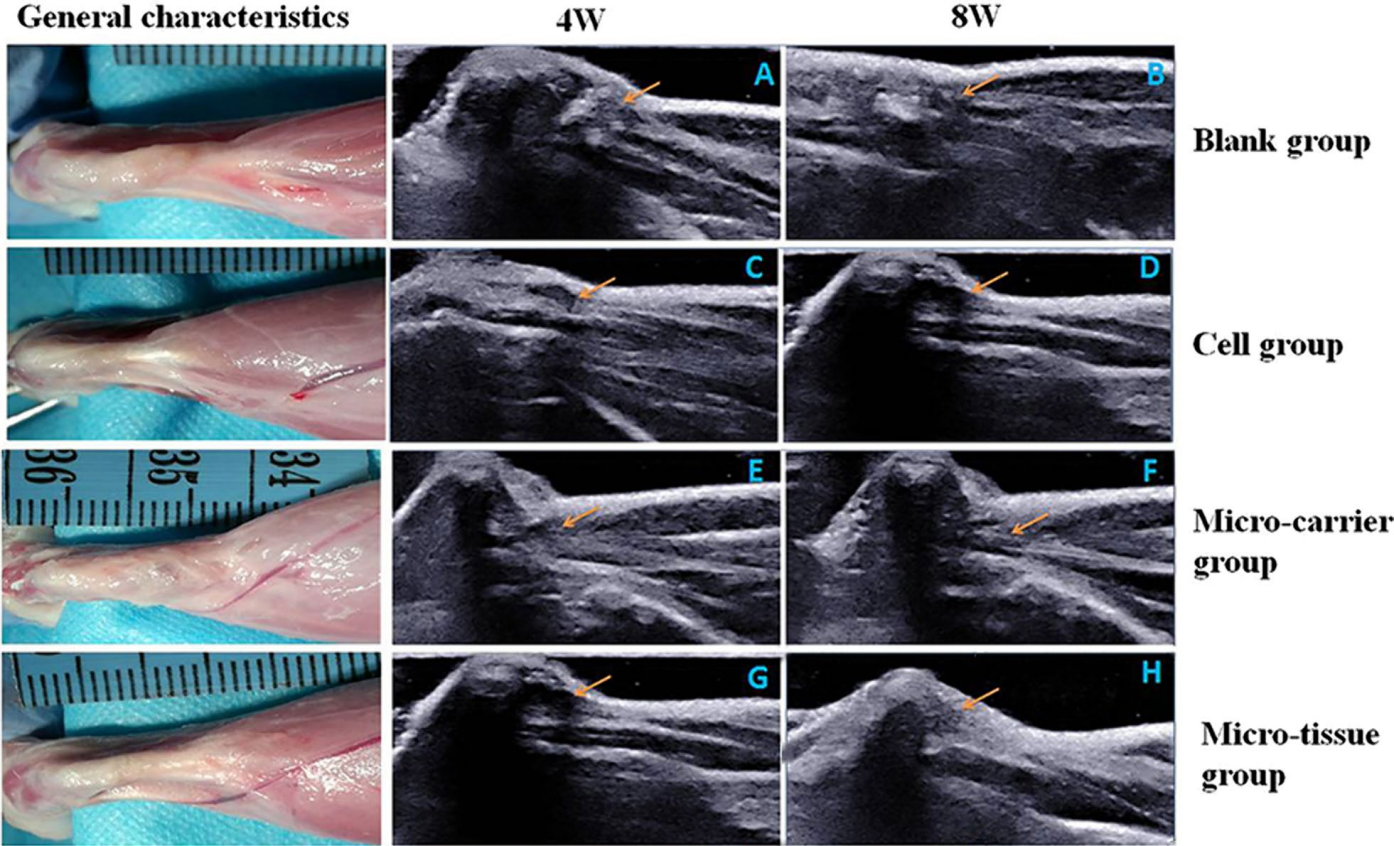
General characteristics at 8 weeks and ultrasound findings. (**A-H**) Ultrasound findings at 4 and 8 weeks. (Orange arrow indicates Achilles tendon)

At 4 weeks and 8 weeks post-surgery, the rats in each group underwent ultrasound examination, during which the continuity, echo intensity and fiber structure of the tendon were observed to evaluate the repair of the Achilles tendon. At 4 weeks, the continuity of the Achilles tendon in the blank group (Fig. 5A) was interrupted, and the two severed ends had retracted to form an empty cavity. Numerous hematomas were seen at the fracture site. In the cell group (Fig. 5C), there was a continuous interruption in the Achilles tendon, with small areas of low echo between the striated areas of high-echo. The broken ends of the tendon were always of low echo or anechoic and numerous hematomas were seen in the tissue. The micro-carrier group showed areas of continuous disruption in the Achilles tendon (Fig. 5E). Passive extension of the dorsal ankle resulted in widened zones of low echo and no echo. When the metatarsal ankle and the triceps of the calf were pressed together, the two ends had a tendency to move closer to each other. In the micro-tissue group (Fig. 5G), the continuity of the Achilles tendon was acceptable and better than in the blank and cell groups. The volume of the tendon increased slightly, but the contour was blurred. There was no obvious hematoma. At 8 weeks, the blank group (Fig. 5B) contracture of the broken ends persisted, with an obvious gap characterized by an uneven low-echo or anechoic area. The Achilles tendon was attached to the surrounding tissue. Achilles tendon repair in the cell group (Fig. 5D) had improved, but tendon continuity was still poor. The damage was still visible and the irregular hypoechoic gap remained, but areas of hematoma were no longer visible. The continuity of the Achilles tendon in the micro-carrier group (Fig. 5F) was acceptable. There was no obvious terminal contracture and the gap was filled with scar tissue that showed little or no echo. The micro-tissue group (Fig. 5H) still showed significant transection of the Achilles tendon, but the tissue continuity was better and the tissues were arranged in an orderly manner. The ultrasound showed parallel bands of strong echo and some thin bands of weak echo.

### Evaluation of tendon repair after transplantation

**Fig. 6:**
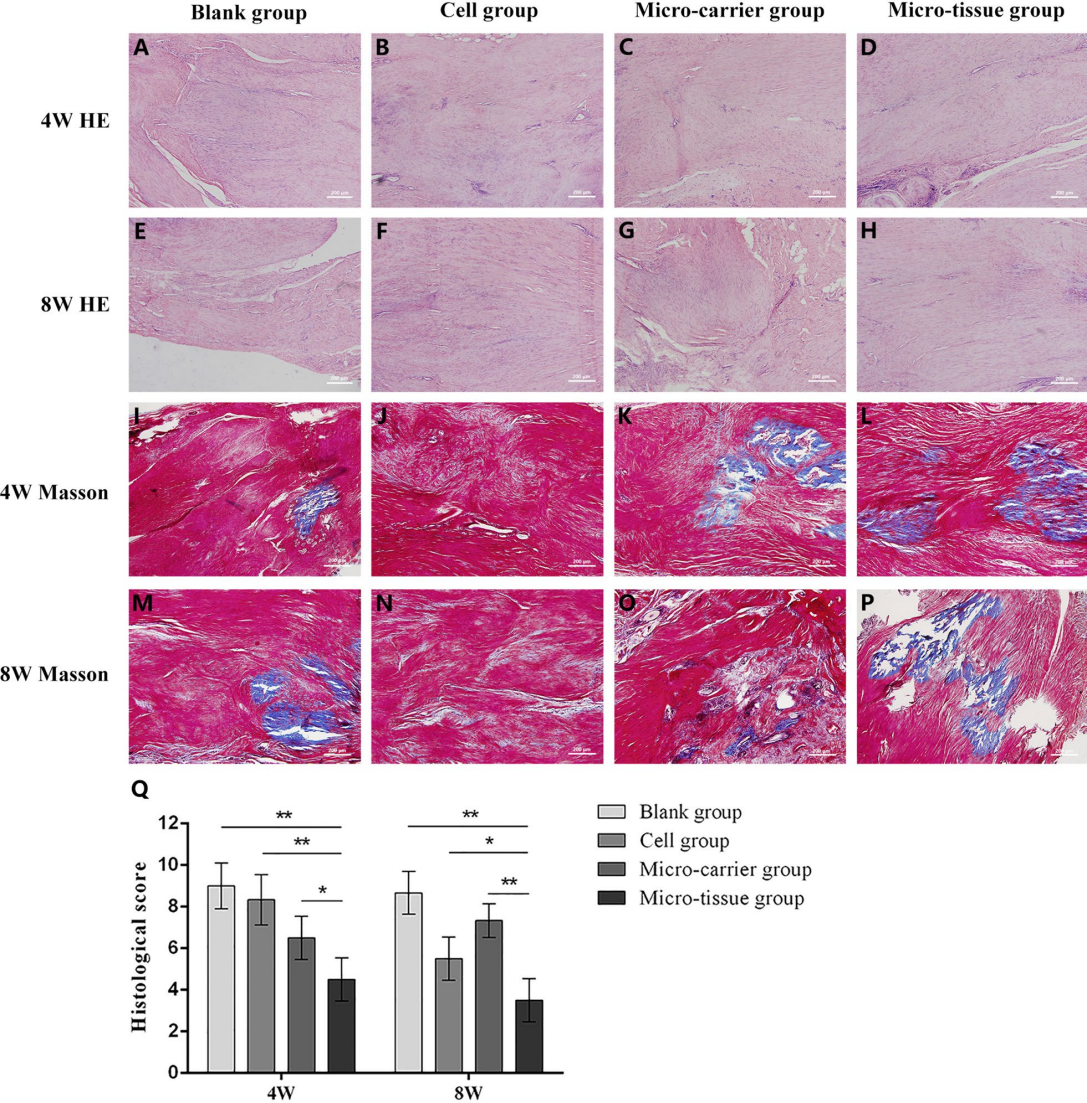
Results of histological staining and histological score. **A**–**H** HE staining. **I**–**P**. Masson trichrome staining. (magnification × 100, scale bar = 200 μm). **Q** Histological score (*p < 0.05 and **p < 0.01)

Histological staining and histological score were used to evaluate the repair effect after tissue-engineered tendon transplantation. The results of HE staining showed that the tissues in the blank (Fig. 6A) and cell groups (Fig. 6B) were disordered in their arrangement, with a large number of inflammatory cells infiltrating the defect and causing tissue edema, and an extensive neovascularization occurred at 4 weeks post-surgery. The collagen fibers in the micro-carrier group (Fig. 6C) were loosely arranged, with elliptical fibrocytes. Inflammatory cell infiltrates and neovascularization were observed without obvious tissue edema. The tissue of the micro-tissue group (Fig. 6D) was relatively well ordered. Inflammatory cells and neovascularization were rare and collagen fiber proliferation was observed. At 8 weeks, there was less inflammatory cell infiltration in all groups. The fibrous structure in the blank (Fig. 6E) and micro-carrier groups (Fig. 6G) was disordered, with visible scar tissue formation. A large number of infiltrating inflammatory cells and neovascularization remained in the cell group (Fig. 6F). The tissue structure was slightly disturbed, with little proliferation of fibrous tissue. In the micro-tissue group (Fig. 6H), a large number of densely arranged collagen fibers were seen along with mature fibrocytes. The nuclei were spindle-shaped and there were few inflammatory cells.

The results of Masson trichrome staining showed that severe necrosis was observed in the anastomotic area in both the blank group (Fig. 6I) and the cell group (Fig. 6J), along with intense inflammatory cell infiltration and angiogenesis at 4 weeks post-surgery. The tissue was disorganized. In the micro-carrier group (Fig. 6K), some collagen fibers were seen, the tissues were loosely arranged but disordered in structure, and inflammatory cells infiltration was obvious. The tissue in the micro-tissue group (Fig. 6L) had an orderly arrangement, and collagen fibers were clearly visible, along with a small inflammatory cell infiltration and angiogenesis. At 8 weeks, there was less inflammatory cell infiltration in each group. Scar tissue formation was clearly evident in the blank (Fig. 6M) and micro-carrier groups (Fig. 6O), with low proliferation of collagen fibers and disordered fiber structure. There were no new collagen fibers in the cell group (Fig. 6N), but inflammatory cell infiltration was evident, although neovascularization was rare. There was significant formation of collagen fibers in the micro-tissue group (Fig. 6P). The tissue structure was ordered and the infiltration of inflammatory cells was less. The results of histological score showed that the histological score in the micro-tissue group was significantly lower than the other three groups at 4 and 8 weeks post-surgery (*p* < 0.05) (Fig. 6Q).

### Biomechanical results

**Fig. 7:**
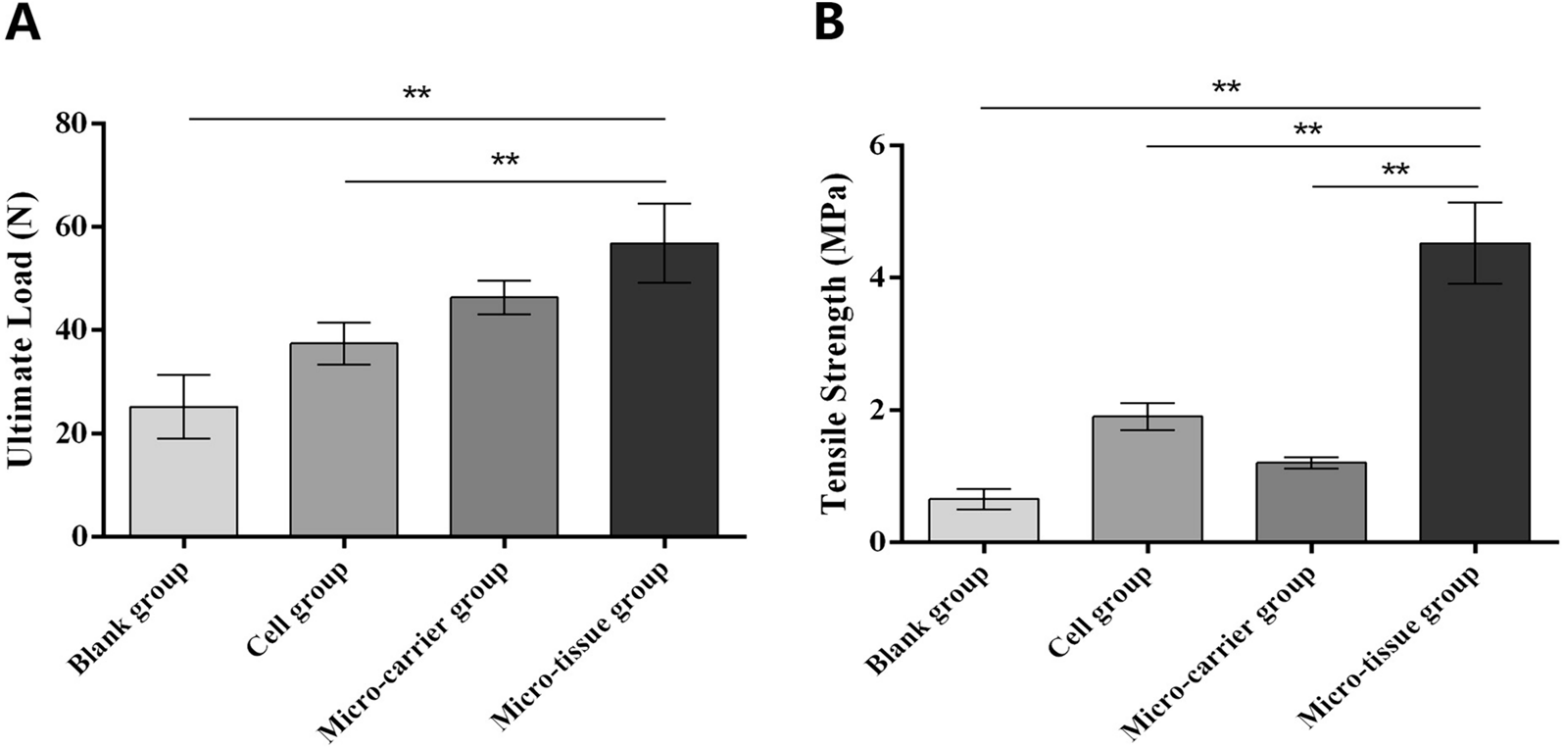
Results of biomechanical analysis. **A** The ultimate load in different groups at 8 weeks after repair. **B** The tensile strength in different groups at 8 weeks after repair (**p < 0.01)

The results of the biomechanical test showed that the ultimate load in the micro-tissue group was significantly higher than that in the blank and cell groups (*p* < 0.01), but the difference was not significant compared to the micro-carrier group (*p* > 0.05) (Fig. 7A). The tensile strength of the micro-tissue group was superior to that of the other three control groups, and the results were statistically different (*p* < 0.01) (Fig. 7B).

## Discussion

Functional repair of injured tendons remains a major challenge for clinical orthopedics. Tendon healing is a complex biological process. Due to low metabolism, low cellularity, and limited vascularization, tendon tissue has an inadequate ability to synthesize extracellular matrix to promote self-repair. Clinically, both conservative and surgical treatments often result in scarring and fibrosis, accompanied by deterioration of biomechanical properties and promotion of tissue adhesion [26]. Therefore, recurrence of tendon ruptures and chronic degenerative tendinopathies often occur in clinical practice [6]. New approaches are desired when repairing tendon injuries. The development of tissue engineering provides new methods for the functional repair of tendons.

Tendon tissue engineering includes seed cells, bioactive factors and, most importantly, scaffold materials. Appropriate scaffolds not only maintain the biomechanical properties of the tendon, but also provide a good environment for seed cell growth and proliferation [27]. Accordingly, biologically derived materials such as ECM are currently used in tissue engineering, with TECM being used in the repair of injured tendons [28]. ECM is a complex macromolecular structure that occupies the space between cells and is mainly responsible for signal transduction between cells, regulating growth and proliferation, and providing mechanical support [29]. The TECM is rich in matrix protein, which promotes seed cell proliferation and differentiation towards tendon tissue while maintaining the stem cell phenotype and inhibiting heterotopic ossification [30]. Furthermore, TECM significantly improves the mechanical properties of the repaired tissue while controlling the repair process of the injured tendon by inhibiting or activating the matrix metalloproteinases of seed cells [14].

In this experiment, we combined TECM with ADSCs to repair Achilles tendon defects in rats. RT-PCR, gait analysis, ultrasound testing, histological analysis, and biomechanical testing were performed to evaluate Achilles tendon repair. At 2 weeks post-surgery, the PCR results in the micro-tissue group were significantly better than those of the other groups, showing high expression of Col I, Col III and TNMD and low VEGF expression in the micro-tissue group. There were little differences in FBN expression between the cell and micro-carrier groups. During the inflammatory phase, endogenous fibroblasts continued to proliferate and differentiate and exogenous fibroblasts migrated into the lesion. Together, these cells synthesized new ECM, collagen fibers, and mucopolysaccharides. Col I is the main component of collagen fibers and is mainly responsible for the mechanical properties of the tendon. This explains the higher ultimate load of the micro-tissue group than that of the other groups. Col III only plays a key role in the early healing process of injured tendons and quickly interweaves into a network structure that is important for the stability of the tendon ends [31, 32]. TNMD is a genetic marker of mature tendons and ligaments and its expression is sensitive to mechanical signals. As an integral part of the tendon tissue, TNMD plays a crucial role in the function of the tendon tissue and maintains the coordination of the function and structure of the tendon tissue. In studies on the mechanical properties of TNMD knockout mice compared to wild-type mice, the mechanical properties of the knockout group were significantly worse than those of the wild-type group and worsened with training [33]. VEGF is a glycoprotein that, upon binding to its receptors, modulates angiogenesis and the permeability of vascular endothelial cells [34, 35]. It is highly expressed during inflammation and is a marker of the tissue inflammatory response. Accordingly, there was little inflammatory response in the micro-tissue group two weeks after surgery. FBN expression was similar in each group, reflecting endogenous and exogenous repair of the injured tendon [36, 37]. Although FBN expression was abundant in all groups, it was still significantly higher in the micro-tissue group than in the blank group. The pathological results showed better continuity in the micro-tissue group, with a large number of densely arranged, ordered collagen fibers. Mature fibroblasts with a spindle-shaped nucleus and a small amount of inflammatory cell infiltrates were observed. Macroscopically, the general characteristics, mechanical properties, gait parameters and ultrasound results of the micro-tissue group were significantly better than those of the other groups, which provided visible evidence of the better repair of the Achilles tendon in the micro-tissue group than other groups.

ADSCs are one of the most commonly used seed cells in tendon tissue engineering [38]. They are easy to obtain, cause minimal harm to the donor, and proliferate massively in vitro within a short period of time while maintaining their phenotype. Similar to tenocytes, uninduced ADSCs secrete collagen type I under general culture conditions [39, 40]. Transmission electron microscopy studies have shown that the cells are rich in rough endoplasmic reticulum, indicating strong maintenance of secretion under co-culture conditions [41]. Therefore, ADSCs are an ideal source of seed cells for tendon tissue engineering due to their easy isolation, multiple differentiation potential and excellent responsiveness to microenvironmental stimuli [42]. While in some cases, ADSCs could be induced to differentiate into tenocytes in vitro, this has yet to be achieved in vivo. However, ADSCs cultured in vitro can differentiate into tenocytes in vivo, which may reflect differences in biological factors in the microenvironment. Therefore, the microenvironment of the seed cells is particularly important in tendon tissue engineering. In this study, seed cells were obtained for use in the simulated tendon tissue microenvironment using decellularized rat tail tendons. The resulting TECM consisted mainly of proteoglycans with large molecular weight and high hydrophilicity, so that the water content of the connective tissue and the exchange of substances between tissues were preserved [43]. In addition, ECM contains a large number of cytokines. These biologically active polypeptides or glycoproteins include IGF, FGF, PDGF, VEGF, and TNMD (44–46). The coordinated action of these cytokines promotes tendon growth and pathological repair.

In this study, we provided a feasible approach to repair Achilles tendon injuries through rat tail tendon extracellular matrix and the combination of ADSCs to produce biological scaffolds that show good repair results. However, this tissue engineering strategy for Achilles tendon repair still poses numerous challenges. For example, how local inflammatory microenvironments and growth factors regulate the differentiation of ADSCs needs to be further investigated. The mechanical properties of the regenerated tissue influence the repair of the Achilles tendon. Therefore, the integration process and regulatory mechanism between biomaterial scaffold and tendon injury site also need to be investigated. Immune rejection of transplants is also a problem that cannot be ignored. The source of the extracellular matrix of human tendons requires further investigation. These are important issues that impact future translation from basic science to clinical application.

## Conclusion

In conclusion, repairing the injured Achilles tendon is a complex process and the treatment is associated with a high risk of complications. In our study, SDS-treated TECM obtained by repeated freezing and thawing yielded a preparation with a very small amount of cell residue and low immunogenicity. TECM has good biocompatibility but no cytotoxicity. When co-culture with ADSCs to repair injured Achilles tendons in rats, TECM promoted the effective healing of injured rat Achilles tendons.

## Data Availability

The datasets used and/or analysed during the current study are available from the corresponding author on reasonable request.
